# It’s time to measure the long-term effects of occupational therapy interventions

**DOI:** 10.1177/03080226231172628

**Published:** 2023-06-05

**Authors:** Edward Duncan

**Affiliations:** Nursing Midwifery and Allied Health Professions Research Unit, Faculty of Health Sciences and Sport, University of Stirling, Stirling, Scotland, UK

In 2021, The Royal College of Occupational Therapists and the James Lind Alliance released their top ten research priorities for occupational therapy (Watson et al., 2021). Their fourth priority highlighted an important and under-researched question: what are the long-term effects of occupational therapy interventions? Despite occupational therapy research developing exponentially over the last 20 years, surprisingly little research has been undertaken to investigate the long-term benefits of many occupational therapy interventions. In our current economically challenging health landscape, consideration needs to be given to de-implementing interventions that do not demonstrate a sustained difference in people’s lives. Therefore, now more than ever, evaluating the long-term benefits of new and established occupational therapy interventions is crucial to understand their impact on people’s quality of life over time.

Conducting high-quality long-term follow-up intervention studies is challenging (Signorell et al., 2022; Wilcox and Ely, 2019). Higher levels of research funding are required to follow up participants over a longer period of time. Participant attrition is considerable in any long-term outcome study as people often drop out of long-term studies and are lost to follow-up. This in turn can lead to smaller sample sizes, diminished returns, limiting and potentially biasing study findings. Strategies to reduce study attrition, such as clear communication of expectations, study incentives such as gift cards, cash or other rewards, personalised communication and follow-up of participants, positive reinforcement and collaboration with participants in the study design have all been shown to help reduce study attrition (Gillies et al., 2021) and should be employed in long-term effectiveness studies. Measuring outcomes over a long period of time can be difficult, as some outcomes change gradually, while others may change rapidly. Finally, long-term data management can become increasingly complex and it can be challenging to ensure that data are collected, stored, and analysed correctly. Many of these challenges can be overcome with advice from clinical trial units and experienced methodologists, who are essential collaborators in any study of this nature. However, preceding all of these challenges is a more theoretical question that should be answered by all occupational therapists who are designing or implementing an intervention: How will this intervention produce the sustained outcomes it aims to? Meaningfully answering this question will enable occupational therapists to articulate how they understand their interventions work, support research funding applications to measure the intervention’s impact, and guide the intervention’s long-term evaluation. Not all occupational therapists are required to understand the methodological challenges of studying the long-term effects of occupational therapy interventions. However, all occupational therapists should be able to articulate the theory of how they believe their interventions will achieve the long-term outcomes they envisage.

**Figure 1.**
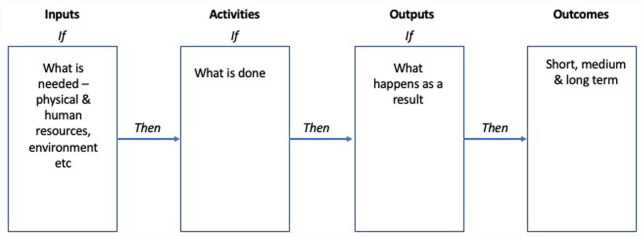
A basic Logic Model.

Given the challenges of studying long-term effects of occupational therapy interventions, it is important to carefully consider if a specific intervention merits such investigation. Having decided your research question is important (Taylor et al., 2023), an important next step is to articulate how you believe an occupational therapy intervention will achieve its intended outcomes. The latest version of the ‘Framework for the development and evaluation of complex interventions’ proposes the development of a Logic Model as a useful way to communicate how an intervention is believed to work (Skivington et al., 2021). Logic Models at their simplest are linear pathways that provide visual representations of the key stages of an intervention, including inputs (what you need to deliver the intervention), activities (what you do during the intervention), outputs (what happens as a result) and outcomes (what are the immediate, medium- and long-term impacts of the intervention). In providing a clear model of how an intervention is believed to work, Logic Models provide a simplified series of ‘If-Then’ relationships that guide and support the evaluation of the intervention and the long-term follow-up of its outcomes. Various Logic Model formats exist, with greater or lesser complexity. Some Logic Models have been developed to illustrate complex causal pathways, while others illustrate an intervention’s unintended or negative consequences (Skivington et al., 2021). A basic linear Logic Model is presented in Figure 1. Examples of Logic Models of varying complexity are easily located on the internet and can be adapted to suit the needs of a specific intervention or programme. Some researchers have already developed published Logic Models of some occupational therapy interventions (Boey and Warren, 2019; Roelke et al., 2022). However, the development of Logic Models to support occupational therapy interventions should become much more routine as a means to articulate how each specific intervention is believed to work. These models can then form the basis for the development of longer-term evaluation.

Long-term follow-up studies of the effectiveness of occupational therapy interventions are needed now more than ever. Delivering these studies is challenging and requires expert teams of researchers with sufficient funding. In a challenging funding landscape, where research awards are highly competitive careful consideration needs to be given to identifying the clinical areas that merit such investment and are most likely to have longer-term impacts. Deciding the research question and having a clearly articulated Logic Model of how the intervention is believed to work are important early steps in this process.
